# Caregiver burden and malnutrition in patients with HIV-associated lymphoma: indirect pathways via symptom distress and family closeness, and a test of resilience as a mediator

**DOI:** 10.3389/fnut.2026.1921747

**Published:** 2026-09-28

**Authors:** Tingting Liu, Jing Lv, Luxiang Xu, Yu Peng, Chaoyu Wang, Xiaomei Zhang, Qing Xiao, Jun Li, Zhaoli Zhang, Yao Liu

**Affiliations:** 1 Department of Hematology-Oncology, Hematologic Oncology Intelligent Diagnosis and Treatment Engineering Research Center of Chongqing Education Commission of China, Chongqing University Cancer Hospital, Chongqing, China; 2 Nursing Department, Chongqing Key Laboratory of Translational Research for Cancer Metastasis and Individualized Treatment, Chongqing University Cancer Hospital, Chongqing, China

**Keywords:** caregiver burden, family closeness, HIV-associated lymphoma, malnutrition, psychosocial factors, resilience, symptom distress

## Abstract

**Background and objective:**

Malnutrition is a frequent and clinically significant complication in patients with HIV-associated lymphoma, adversely affecting treatment tolerance, prognosis, and quality of life. Although disease-related and immunological determinants of malnutrition have been widely reported, the potential psychosocial pathways—particularly the role of caregiver burden—in patients with HIV-associated lymphoma remain insufficiently characterized. This study aimed to identify key determinants of malnutrition and to examine whether caregiver burden exerts indirect effects through symptom distress and psychosocial factors.

**Methods:**

This single-center cross-sectional study included 200 patients with HIV-associated lymphoma and their family caregivers. Nutritional status was assessed using the Patient-Generated Subjective Global Assessment (PG-SGA), with the total score analyzed as a continuous outcome. Symptom burden, caregiver burden, psychological resilience, and family closeness were assessed using standardized instruments. Univariable and multivariable linear regression analyses were conducted using HC3 heteroscedasticity-consistent inference. Indirect statistical associations were examined using bootstrap analyses, followed by exploratory subgroup and sensitivity analyses.

**Results:**

Higher symptom burden and IPI risk were associated with higher PG-SGA total scores, whereas higher CD4 + T-cell counts and serum albumin concentrations were associated with lower scores (all *p* ≤ 0.005). Age, sex, and caregiver burden were not independently associated with PG-SGA score. Exploratory analyses provided no statistically supported evidence of an indirect association through symptom burden or of differences by age, sex, or CD4 + T-cell count. Sensitivity analyses produced consistent conclusions.

**Conclusion:**

Nutritional status in patients with HIV-associated lymphoma was associated mainly with symptom burden, immune status, serum albumin, and lymphoma prognostic risk. Caregiver burden showed neither an independent association with PG-SGA score nor a confirmed indirect association through symptom burden. Larger multicenter longitudinal studies are needed to clarify the temporal relationships among caregiver burden, psychosocial factors, and nutritional status.

## 1 Introduction

Malnutrition is a common yet underrecognized complication among individuals with HIV-associated lymphoma, with reported prevalence rates ranging from 40 to 70%, substantially higher than those observed in non-oncologic populations living with HIV (1, 2). The coexistence of HIV infection and lymphoma-related disease burden exacerbates metabolic dysregulation, immune suppression, and chronic inflammation, accelerating nutritional depletion and physical frailty (3–5). Malnutrition in this population has been consistently associated with poor treatment tolerance, impaired immune recovery, and reduced survival, underscoring the need to clarify its multifactorial determinants (6, 7). Despite its clinical significance, the psychosocial mechanisms contributing to nutritional decline in patients with HIV-associated lymphoma remain insufficiently understood.

Existing studies in HIV-related cancers and lymphoma populations have predominantly emphasized physiological and disease-related factors, including body mass index, serum albumin, CD4 cell count, and disease stage, while psychosocial influences have received comparatively limited attention (8). In real-world clinical contexts, both people living with HIV-associated lymphoma and their primary caregivers are exposed to persistent emotional stressors, stigma, uncertainty regarding prognosis, and caregiving fatigue (9, 10). Elevated caregiver burden may compromise emotional availability and caregiving quality, thereby intensifying patients’ symptom distress, suppressing appetite, and ultimately worsening nutritional status (11, 12).

From a biopsychosocial perspective, chronic psychological stress is known to activate neuroendocrine and inflammatory pathways that disrupt appetite regulation, nutrient absorption, and immune restoration, offering a plausible biological link between psychosocial strain and physiological deterioration (13–15). Although this integrative framework has been proposed in oncology and chronic disease research, empirical evidence supporting its applicability in patients with HIV-associated lymphoma remains scarce.

We hypothesized that caregiver burden would be indirectly associated with poorer nutritional status in patients with HIV-associated lymphoma through symptom burden and related psychosocial factors, including family closeness and psychological resilience. Accordingly, this study integrated clinical, immunological, nutritional, and psychosocial data to identify factors associated with PG-SGA total score and to evaluate the proposed indirect statistical associations. Exploratory subgroup analyses were conducted according to age, sex, and CD4 + T-cell count, together with sensitivity analyses evaluating the robustness of the findings. By examining the relationships among caregiver burden, symptom burden, psychosocial characteristics, and nutritional status, this study aimed to inform a broader biopsychosocial understanding of malnutrition in patients with HIV-associated lymphoma.

## 2 Methods

### 2.1 Study design and setting

This cross-sectional observational study was conducted at a tertiary specialized hospital in Chongqing, China. Patients diagnosed with HIV-associated lymphoma and their caregivers underwent a one-time assessment of nutritional, and psychosocial variables. Relevant clinical, laboratory, and anthropometric data were additionally extracted from the patients’ hospital medical records. A total of 200 patient–caregiver dyads with complete data required for the primary analyses were included in the final analysis. The study aimed to investigate factors associated with poorer nutritional status and to explore the hypothesized indirect statistical associations among caregiver burden, patient symptom burden, psychosocial factors, and nutritional status in this patient population. Because all principal study variables were assessed cross-sectionally, the analyses were not intended to establish temporal or causal relationships. The study was reported in accordance with the Strengthening the Reporting of Observational Studies in Epidemiology (STROBE) guidelines.

### 2.2 Participants

Eligible participants were adults (≥18 years) with laboratory-confirmed HIV infection and histopathologically diagnosed lymphoma who were clinically stable and able to complete all questionnaires. Exclusion criteria included: (1) severe psychiatric or cognitive disorder; (2) acute opportunistic infection or severe metabolic disease; (3) recent (within 1 month) parenteral nutrition or rapid body weight fluctuation; and (4) incomplete clinical or questionnaire data.

A total of 236 patients were screened for eligibility, of whom 211 met the inclusion criteria, and 200 completed all assessments and were included in the final analysis (Figure 1). Consecutive sampling and standardized data collection procedures were employed to minimize selection bias. Missing data (<5%) were handled through listwise deletion, and sensitivity analyses confirmed that their exclusion did not materially affect the results.

**Figure 1:**
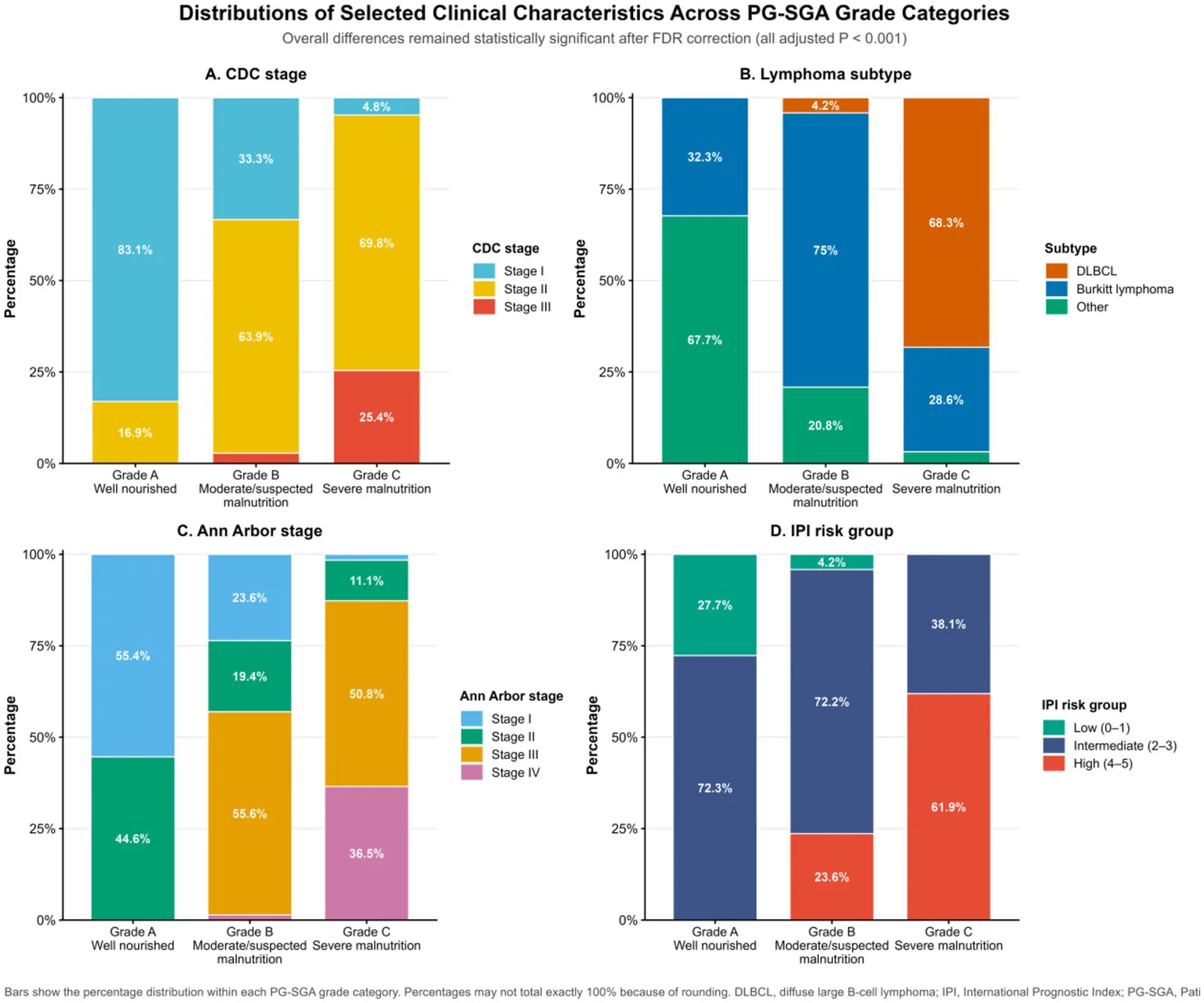
Distributions of selected clinical characteristics across PG-SGA grade categories. Percentage-stacked bar charts show the distributions of **(A)** CDC stage, **(B)** lymphoma subtype, **(C)** Ann Arbor stage, and **(D)** IPI risk group among participants classified as well nourished (PG-SGA grade A, *n* = 65), moderately or suspected of being malnourished (grade B, *n* = 72), and severely malnourished (grade C, *n* = 63). Percentages were calculated within each PG-SGA grade category and may not total exactly 100% because of rounding. Overall differences were assessed using Fisher’s exact test with Monte Carlo simulation based on 10,000 replicates. All four variables differed across the PG-SGA grade categories after Benjamini–Hochberg false discovery rate correction (all adjusted *p* < 0.001). These comparisons indicate statistical associations and do not establish temporal or causal relationships. DLBCL, diffuse large B-cell lymphoma; IPI, International Prognostic Index; PG-SGA, Patient-Generated Subjective Global Assessment.

Sample size was estimated *a priori* using G*Power 3.1, assuming a medium effect size (*f*^2^ = 0.15), α = 0.05, and power = 0.80, which required at least 138 participants; the final sample size provided adequate statistical power for all analyses.

### 2.3 Measures

Nutritional status was assessed using the Patient-Generated Subjective Global Assessment (PG-SGA), which incorporates weight change, dietary intake, nutrition-impact symptoms, functional capacity, disease-related factors, and physical examination findings. Higher total scores indicate poorer nutritional status. The PG-SGA total score was treated as the primary nutritional outcome and analyzed as a continuous dependent variable. Body mass index (BMI) was considered a separate anthropometric indicator and was not used as an equivalent measure of PG-SGA-defined nutritional status. PG-SGA grade, which is derived from the PG-SGA assessment, was not included as a predictor of the PG-SGA total score.

Caregiver burden was assessed using the 22-item Zarit Burden Interview (ZBI), with total scores ranging from 0 to 88; higher scores indicate greater caregiver burden (Cronbach’s α = 0.93 in the present study). Patient symptom burden was evaluated using the MD Anderson Symptom Inventory (MDASI), which comprises 13 symptom severity items and 6 symptom-interference items rated on an 11-point scale from 0 to 10 (Cronbach’s α = 0.94 in the present study). Psychological resilience was assessed using the 25-item Connor–Davidson Resilience Scale (CD-RISC), with higher total scores indicating greater psychological resilience. Family closeness was assessed using the cohesion subscale of the Family Adaptability and Cohesion Evaluation Scales, second edition (FACES-II), with higher scores indicating greater family closeness.

Clinical, laboratory, and anthropometric variables included Ann Arbor stage, International Prognostic Index (IPI), CD4 + T-cell count (cells/μL), serum albumin (g/L), body mass index (BMI), percentage of body weight loss during the preceding 3 months, and duration of HIV infection (years). The IPI score was extracted directly from the hospital medical records. The conventional IPI comprises five adverse prognostic factors: age >60 years, Ann Arbor stage III or IV disease, serum lactate dehydrogenase above the upper limit of normal, Eastern Cooperative Oncology Group performance status ≥2, and involvement of more than one extranodal site. One point is assigned for each factor, yielding a total score ranging from 0 to 5. Because the individual components used to derive the recorded IPI score were not available in the study database, the score was not recalculated or independently verified by the investigators. The conventional four risk groups are low risk (0–1), low-intermediate risk (2), high-intermediate risk (3), and high risk (4–5). For the present analysis, the low-intermediate and high-intermediate groups were combined to form three study-defined categories: low (0–1), intermediate (2–3), and high (4–5). The IPI was entered into the regression models as a three-category variable. All clinical, laboratory, and anthropometric data were obtained from hospital medical records. All laboratory and anthropometric data were obtained from hospital medical records.

### 2.4 Statistical analysis

All analyses were performed using R software (version 4.3.2, R Foundation for Statistical Computing, Vienna, Austria). Continuous variables were described as mean ± standard deviation (SD) or median (interquartile range, IQR), and categorical variables as frequency and percentage.

Univariate linear regression models examined associations with PG-SGA total score, with FDR correction applied across 11 variable-level tests. The multivariable model included age, sex, MDASI total score, CD4 + T-cell count, serum albumin, IPI risk group, and ZBI total score, prespecified according to clinical relevance and the conceptual framework. CD4 + T-cell count was scaled per 100 cells/μL. PG-SGA grade was excluded because it was derived from the PG-SGA assessment. HC3 heteroscedasticity-consistent standard errors, 95% confidence intervals, and *p*-values were reported.

Multicollinearity was assessed using variance inflation factors. Model diagnostics included residual-versus-fitted, normal Q–Q, and scale–location plots, the Breusch–Pagan and Shapiro–Wilk tests, and Cook’s distance. Observations with Cook’s distance >4/n were retained in the primary analysis and examined in a sensitivity analysis.

Exploratory parallel mediation analyses evaluated indirect statistical associations between caregiver burden and PG-SGA total score through symptom burden, family closeness, and psychological resilience. The adjusted model included age, sex, CD4 + T-cell count, serum albumin, and IPI risk group. Specific and total indirect associations were estimated using 5,000 bootstrap resamples and reported with percentile bootstrap 95% confidence intervals.

Sensitivity analyses compared the unadjusted model, the primary covariate-adjusted model, an expanded model additionally adjusted for BMI and duration of HIV infection, and a model excluding observations that exceeded the Cook’s distance screening threshold of 4/n. All indirect associations were estimated using 5,000 bootstrap resamples. All statistical tests were two-sided, with *p* < 0.05 considered statistically significant unless FDR-adjusted *p*-values were specified.

### 2.5 Ethical considerations

The study protocol was reviewed and approved by the Institutional Review Board of Chongqing University Cancer Hospital (approval No. CZLS2023028-A). As this research involved only secondary analysis of de-identified data and posed no more than minimal risk to participants, the IRB granted a waiver of the requirement for informed consent. The study was conducted in full compliance with the ethical principles of the Declaration of Helsinki. All data were de-identified and maintained with strict confidentiality to safeguard the privacy of patients and their caregivers.

## 3 Results

### 3.1 Socio-demographic and clinical characteristics of the participants

Baseline characteristics of the 200 patients with HIV-associated lymphoma are summarized in Table 1. Participants were middle-aged (mean 44.26 ± 8.97 years) with a median HIV duration of 11 years (IQR: 6–15) and a mean CD4 count of 411.54 ± 150.81 cells/μL, indicating moderate immune suppression.

**Table 1:** Socio-demographic and clinical characteristics of study participants.

Variable	Total (*N* = 200)^1^
Age	
Mean ± SD	44.26 ± 8.97
Median (Q1, Q3)	43.00 (36.00, 51.00)
Min, Max	30.00, 60.00
Years since HIV infection	
Mean ± SD	10.34 ± 5.19
Median (Q1, Q3)	11.00 (6.00, 15.00)
Min, Max	2.00, 18.00
Recent CD4 count	
Mean ± SD	411.54 ± 150.81
Median (Q1, Q3)	384.00 (285.50, 554.00)
Min, Max	79.00, 697.00
IPI score	
1	21 (10.5%)
2	54 (27.0%)
3	69 (34.5%)
4	31 (15.5%)
5	25 (12.5%)
BMI	
Mean ± SD	20.45 ± 4.05
Median (Q1, Q3)	20.06 (17.30, 23.31)
Min, Max	12.65, 31.11
Weight loss within 3 months (%)	
Mean ± SD	11.12 ± 5.40
Median (Q1, Q3)	11.00 (6.40, 15.60)
Min, Max	0.00, 29.00
Serum albumin (g/L)	
Mean ± SD	35.11 ± 9.26
Median (Q1, Q3)	34.35 (28.15, 40.90)
Min, Max	20.00, 57.70
MDASI total score	
Mean ± SD	31.12 ± 9.92
Median (Q1, Q3)	30.00 (23.00, 39.50)
Min, Max	12.00, 51.00
CD-RISC total score	
Mean ± SD	47.27 ± 13.60
Median (Q1, Q3)	46.00 (38.00, 56.50)
Min, Max	12.00, 83.00
Sex	
Male	95 (48%)
Female	105 (53%)
Education level	
Primary school	33 (17%)
Middle school	30 (15%)
High school	34 (17%)
Vocational school	30 (15%)
College	36 (18%)
University	37 (19%)
Marital status	
Single	44 (22%)
Married	49 (25%)
Divorced	57 (29%)
Widowed	50 (25%)
Occupation	
Farmer	35 (18%)
Worker	25 (13%)
Clerk	50 (25%)
Professional	23 (12%)
Business	28 (14%)
Retired	21 (11%)
Unemployed	18 (9.0%)
Place of residence	
Urban	63 (32%)
Suburban	75 (38%)
Rural	62 (31%)
Monthly family income	
<3,000 RMB	43 (29%)
3,000–6,000 RMB	48 (33%)
>6,000 RMB	56 (38%)
Missing	53
CDC stage	
Stage I	81 (41%)
Stage II	101 (51%)
Stage III	18 (9.0%)
Lymphoma pathological type	
DLBCL	46 (23%)
Burkitt	93 (47%)
Other	61 (31%)
Ann Arbor stage	
Stage I	54 (27%)
Stage II	50 (25%)
Stage III	72 (36%)
Stage IV	24 (12%)
IPI score category	
Low (0–1)	21 (10.5%)
Intermediate (2–3)	123 (61.5%)
High (4–5)	56 (28%)

^1^Data presented as mean ± SD, median (IQR), or n (%). BMI, body mass index; CD4, cluster of differentiation 4; CD-RISC, Connor–Davidson Resilience Scale; CDC, Centers for Disease Control and Prevention; DLBCL, diffuse large B-cell lymphoma; HIV, human immunodeficiency virus; IPI, International Prognostic Index; MDASI, MD Anderson Symptom Inventory; Q1, first quartile; Q3, third quartile; RMB, renminbi; SD, standard deviation.

Half of the cohort presented with intermediate IPI risk (2–3 points), while 38 and 13% were classified as low- and high-risk, respectively. More than two-thirds of patients were in Ann Arbor stage II–III, and the predominant pathological subtype was Burkitt lymphoma (47%), followed by diffuse large B-cell lymphoma (23%) and other types (31%).

Nutritional parameters revealed a mean BMI of 20.45 ± 4.05 kg/m^2^ and an average 11.1% body weight loss within 3 months. The mean serum albumin level was 35.11 ± 9.26 g/L, close to the clinical threshold for hypoalbuminemia (<35 g/L), suggesting a high prevalence of nutritional compromise.

Psychological and symptom assessments showed a mean MDASI score of 31.12 ± 9.92, reflecting a moderate-to-high symptom burden, and a CD-RISC score of 47.27 ± 13.60, indicating moderate resilience. Socio-demographic variables were evenly distributed across sex (48% male), education, marital status, and income categories.

Overall, participants demonstrated moderate disease severity, intermediate immune function, and substantial nutritional vulnerability, establishing a clear clinical foundation for subsequent comparisons across nutritional status groups and predictors of malnutrition.

### 3.2 Comparison of characteristics across PG-SGA grade categories

Participants were classified as well nourished (PG-SGA grade A, *n* = 65), moderately or suspected of being malnourished (grade B, *n* = 72), or severely malnourished (grade C, *n* = 63). Comparisons across the three groups are presented in Table 2. Continuous and categorical variables were compared using the Kruskal–Wallis test and Fisher’s exact test with Monte Carlo simulation, respectively. *p*-values were adjusted using the Benjamini–Hochberg false discovery rate procedure.

**Table 2:** Comparison of participant characteristics across PG-SGA grade categories.

Variable	Overall, N = 200^1^	A: Well nourished *N* = 65^1^	B: Moderately/suspected malnutrition *N* = 72^1^	C: Severely malnourished *N* = 63^1^	*P*-value^2^	FDR-adjusted *P-*value^3^
Age, years	43.00 (36.00, 51.00)	42.00 (35.00, 51.00)	43.00 (40.00, 50.00)	46.00 (36.00, 53.00)	0.588	0.893
Sex					0.864	0.902
Male	95 (47.5%)	32 (49.2%)	35 (48.6%)	28 (44.4%)		
Female	105 (52.5%)	33 (50.8%)	37 (51.4%)	35 (55.6%)		
Education level					0.657	0.893
Primary school	33 (16.5%)	11 (16.9%)	13 (18.1%)	9 (14.3%)		
Middle school	30 (15.0%)	9 (13.8%)	11 (15.3%)	10 (15.9%)		
High school	34 (17.0%)	13 (20.0%)	9 (12.5%)	12 (19.0%)		
Vocational school	30 (15.0%)	7 (10.8%)	13 (18.1%)	10 (15.9%)		
College	36 (18.0%)	8 (12.3%)	15 (20.8%)	13 (20.6%)		
University	37 (18.5%)	17 (26.2%)	11 (15.3%)	9 (14.3%)		
Marital status					0.892	0.902
Single	44 (22.0%)	15 (23.1%)	15 (20.8%)	14 (22.2%)		
Married	49 (24.5%)	13 (20.0%)	18 (25.0%)	18 (28.6%)		
Divorced	57 (28.5%)	22 (33.8%)	19 (26.4%)	16 (25.4%)		
Widowed	50 (25.0%)	15 (23.1%)	20 (27.8%)	15 (23.8%)		
Duration of HIV infection, years	11.00 (6.00, 15.00)	10.00 (7.00, 15.00)	11.00 (5.00, 15.00)	12.00 (5.00, 16.00)	0.683	0.893
CDC stage					<0.001	<0.001
Stage I	81 (40.5%)	54 (83.1%)	24 (33.3%)	3 (4.8%)		
Stage II	101 (50.5%)	11 (16.9%)	46 (63.9%)	44 (69.8%)		
Stage III	18 (9.0%)	0 (0.0%)	2 (2.8%)	16 (25.4%)		
CD4 + T-cell count, cells/μL	384.00 (285.50, 554.00)	551.00 (523.00, 566.00)	360.50 (271.50, 561.50)	329.00 (226.00, 364.00)	<0.001	<0.001
Lymphoma subtype					<0.001	<0.001
DLBCL	46 (23.0%)	0 (0.0%)	3 (4.2%)	43 (68.3%)		
Burkitt lymphoma	93 (46.5%)	21 (32.3%)	54 (75.0%)	18 (28.6%)		
Other	61 (30.5%)	44 (67.7%)	15 (20.8%)	2 (3.2%)		
Ann Arbor stage					<0.001	<0.001
Stage I	54 (27.0%)	36 (55.4%)	17 (23.6%)	1 (1.6%)		
Stage II	50 (25.0%)	29 (44.6%)	14 (19.4%)	7 (11.1%)		
Stage III	72 (36.0%)	0 (0.0%)	40 (55.6%)	32 (50.8%)		
Stage IV	24 (12.0%)	0 (0.0%)	1 (1.4%)	23 (36.5%)		
IPI risk group					<0.001	<0.001
Low (0–1)	21 (10.5%)	18 (27.7%)	3 (4.2%)	0 (0.0%)		
Intermediate (2–3)	123 (61.5%)	47 (72.3%)	52 (72.2%)	24 (38.1%)		
High (4–5)	56 (28.0%)	0 (0.0%)	17 (23.6%)	39 (61.9%)		
BMI, kg/m^2^	20.06 (17.30, 23.31)	20.44 (17.65, 23.83)	19.89 (17.62, 23.12)	19.14 (16.61, 23.53)	0.678	0.893
Weight loss during the preceding 3 months, %	11.00 (6.40, 15.60)	10.70 (7.40, 15.20)	11.65 (8.55, 15.65)	10.30 (5.80, 15.50)	0.486	0.893
Serum albumin, g/L	34.35 (28.15, 40.90)	43.00 (39.30, 50.40)	34.30 (28.80, 38.40)	28.50 (23.90, 32.30)	<0.001	<0.001
MDASI total score	30.00 (23.00, 39.50)	29.00 (24.00, 34.00)	23.00 (19.50, 27.00)	44.00 (39.00, 46.00)	<0.001	<0.001
CD-RISC total score	46.00 (38.00, 56.50)	48.00 (39.00, 57.00)	46.00 (37.50, 56.00)	46.00 (37.00, 56.00)	0.850	0.902
family closeness score	51.00 (39.00, 66.00)	44.00 (35.00, 66.00)	52.00 (39.00, 62.50)	57.00 (44.00, 69.00)	0.047	0.101
ZBI total score	42.00 (28.00, 57.00)	46.00 (23.00, 58.00)	42.00 (28.00, 55.00)	38.00 (29.00, 58.00)	0.902	0.902

^1^Median (Q1, Q3); n (%). ^2^Kruskal-Wallis rank sum test; Fisher’s Exact Test for Count Data with simulated p-value (based on 10,000 replicates). ^3^Benjamini and Hochberg correction for multiple testing. BMI, body mass index; CDC, Centers for Disease Control and Prevention; CD4, cluster of differentiation 4; DLBCL, diffuse large B-cell lymphoma; IPI, international prognostic index; MDASI, MD Anderson symptom inventory; CD-RISC, Connor–Davidson resilience scale; ZBI, Zarit burden interview; PG-SGA, patient-generated subjective global assessment; FDR, false discovery rate.

After FDR correction, CDC stage, CD4 + T-cell count, lymphoma subtype, Ann Arbor stage, and IPI risk group differed across the PG-SGA grade categories (all adjusted *p* < 0.001). Grade C included higher proportions of participants with advanced CDC and Ann Arbor stages and high IPI risk, whereas median CD4 + T-cell counts were lower in grade C than in grade A. The distributions of these categorical clinical characteristics are shown in Figure 1.

Serum albumin concentration and MDASI total score also differed across the groups (both adjusted *p* < 0.001). Median serum albumin concentrations were 43.00, 34.30, and 28.50 g/L in grades A, B, and C, respectively. Grade C had the highest median MDASI score, although the scores did not show a monotonic pattern across all three grades. These continuous-variable distributions are shown in Figure 2.

**Figure 2:**
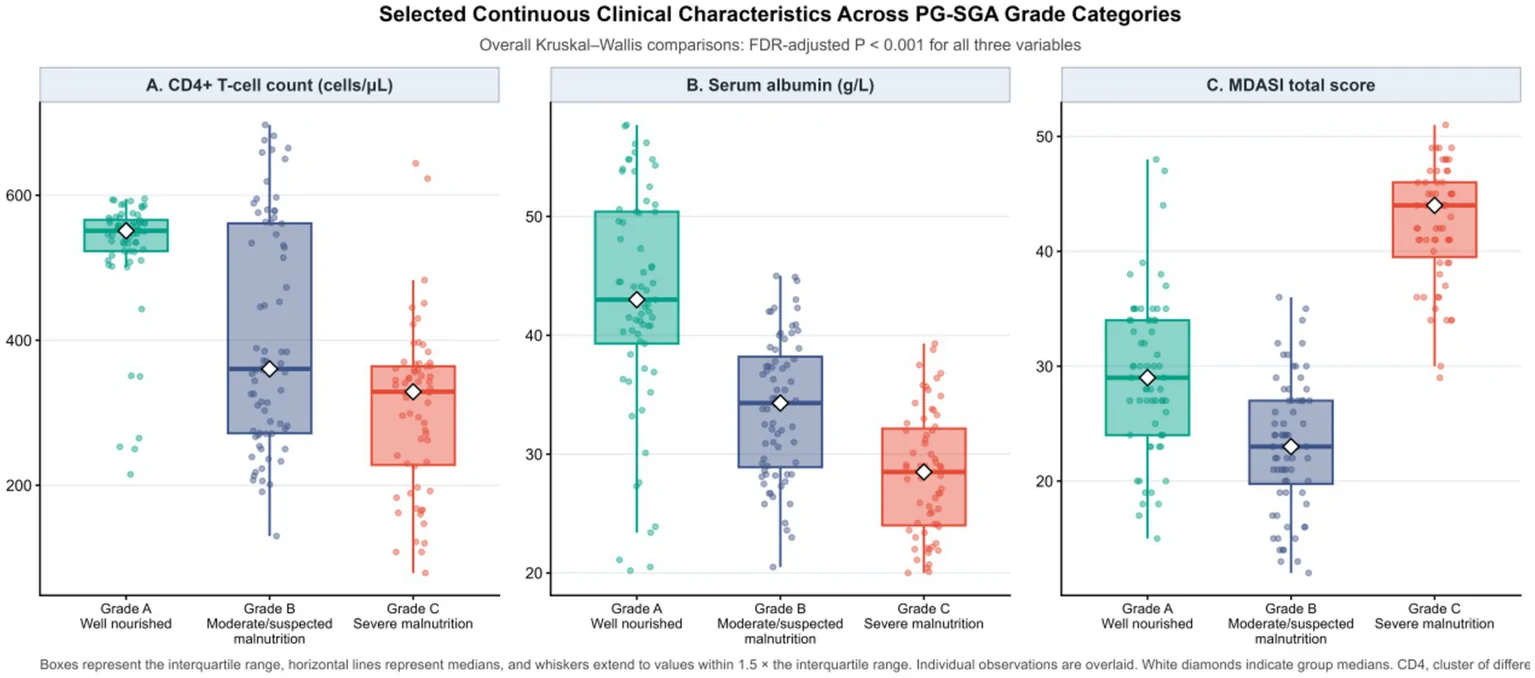
Distributions of selected continuous clinical characteristics across PG-SGA grade categories. Box plots show the distributions of **(A)** CD4 + T-cell count, **(B)** serum albumin concentration, and **(C)** MDASI total score among participants classified as well nourished (PG-SGA grade A, *n* = 65), moderately or suspected of being malnourished (grade B, *n* = 72), and severely malnourished (grade C, *n* = 63). Boxes represent the interquartile range, horizontal lines and white diamonds indicate group medians, and whiskers extend to the most extreme values within 1.5 times the interquartile range. Individual observations are overlaid with horizontal jitter. Overall differences were assessed using the Kruskal–Wallis test. All three variables differed across the PG-SGA grade categories after Benjamini–Hochberg false discovery rate correction (all adjusted *p* < 0.001). Reported *p*-values refer to overall three-group comparisons and do not identify specific pairwise differences. CD4, cluster of differentiation 4; MDASI, MD Anderson Symptom Inventory; PG-SGA, Patient-Generated Subjective Global Assessment.

No statistically significant differences were found in age, sex, education level, marital status, duration of HIV infection, BMI, recent weight loss, CD-RISC total score, or ZBI total score after FDR correction. The unadjusted difference in family closeness scores (*p* = 0.047) was no longer statistically significant after correction (adjusted *p* = 0.101). Overall, the findings indicate cross-sectional associations between PG-SGA grade and selected clinical characteristics, without establishing temporal or causal relationships.

Data are presented as median (Q1, Q3) or n (%), as appropriate. *p*-values were calculated using the Kruskal–Wallis test for continuous variables and Fisher’s exact test with Monte Carlo simulation based on 10,000 replicates for categorical variables. FDR-adjusted *p*-values were calculated using the Benjamini–Hochberg procedure. BMI was treated as an independent anthropometric indicator and was not considered equivalent to nutritional status assessed using the PG-SGA.

### 3.3 Correlation analysis among key study variables

Pairwise Spearman correlations among the nutritional, clinical, and psychosocial variables are presented in Figure 3. After Benjamini–Hochberg FDR correction across all 36 comparisons, the PG-SGA total score was positively correlated with MDASI total score (ρ = 0.47, adjusted *p* < 0.001) and weakly correlated with family closeness score (ρ = 0.19, adjusted *p* = 0.031). Negative correlations were observed between PG-SGA total score and CD4 + T-cell count (ρ = −0.53, adjusted *p* < 0.001) and between PG-SGA total score and serum albumin concentration (ρ = −0.59, adjusted *p* < 0.001). Correlations of PG-SGA total score with ZBI total score, CD-RISC total score, BMI, and recent weight loss were not statistically significant after FDR correction.

**Figure 3:**
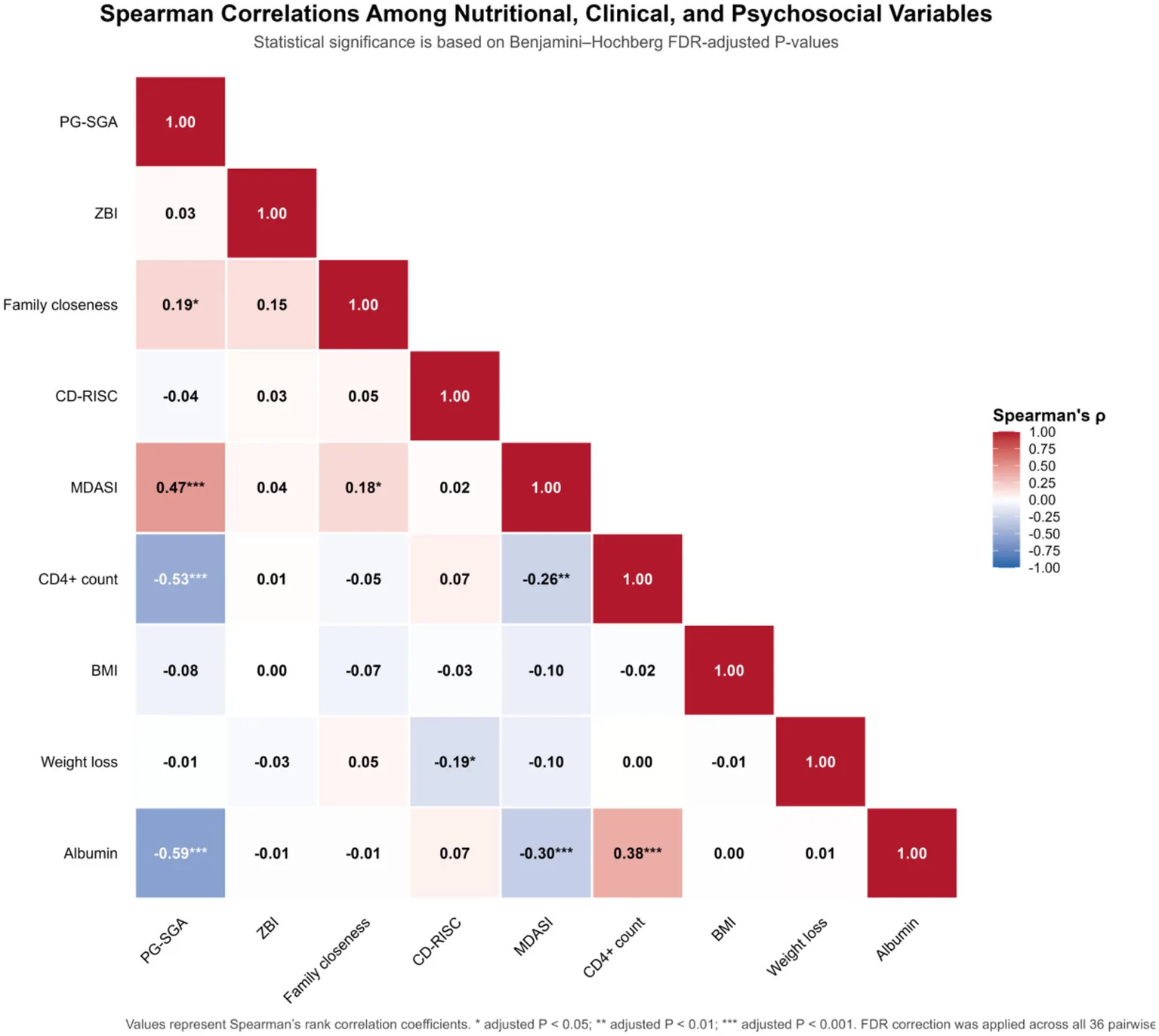
Spearman correlations among nutritional, clinical, and psychosocial variables. The heatmap presents pairwise Spearman rank correlation coefficients (ρ) among PG-SGA total score, ZBI total score, family closeness score, CD-RISC total score, MDASI total score, CD4 + T-cell count, BMI, percentage of body weight loss within the preceding 3 months, and serum albumin concentration. Red and blue cells indicate positive and negative correlations, respectively, with greater color intensity representing a larger absolute correlation coefficient. Statistical significance was evaluated using Benjamini–Hochberg false discovery rate correction across all 36 pairwise comparisons. *Adjusted *p* < 0.05; **adjusted *p* < 0.01; ***adjusted *p* < 0.001. BMI, body mass index; CD4, cluster of differentiation 4; CD-RISC, Connor–Davidson Resilience Scale; FDR, false discovery rate; MDASI, MD Anderson Symptom Inventory; PG-SGA, Patient-Generated Subjective Global Assessment; ZBI, Zarit Burden Interview.

The correlations of PG-SGA total score with the selected study variables, together with bootstrap 95% confidence intervals, are summarized in Figure 4. The largest absolute correlation involving PG-SGA was observed with serum albumin concentration, followed by CD4 + T-cell count and MDASI total score. The bootstrap confidence intervals for these correlations did not include zero. The correlation with family closeness was comparatively weak, despite remaining statistically significant after FDR correction. No statistically significant bivariate correlation was observed between PG-SGA total score and caregiver burden as measured using the ZBI.

**Figure 4:**
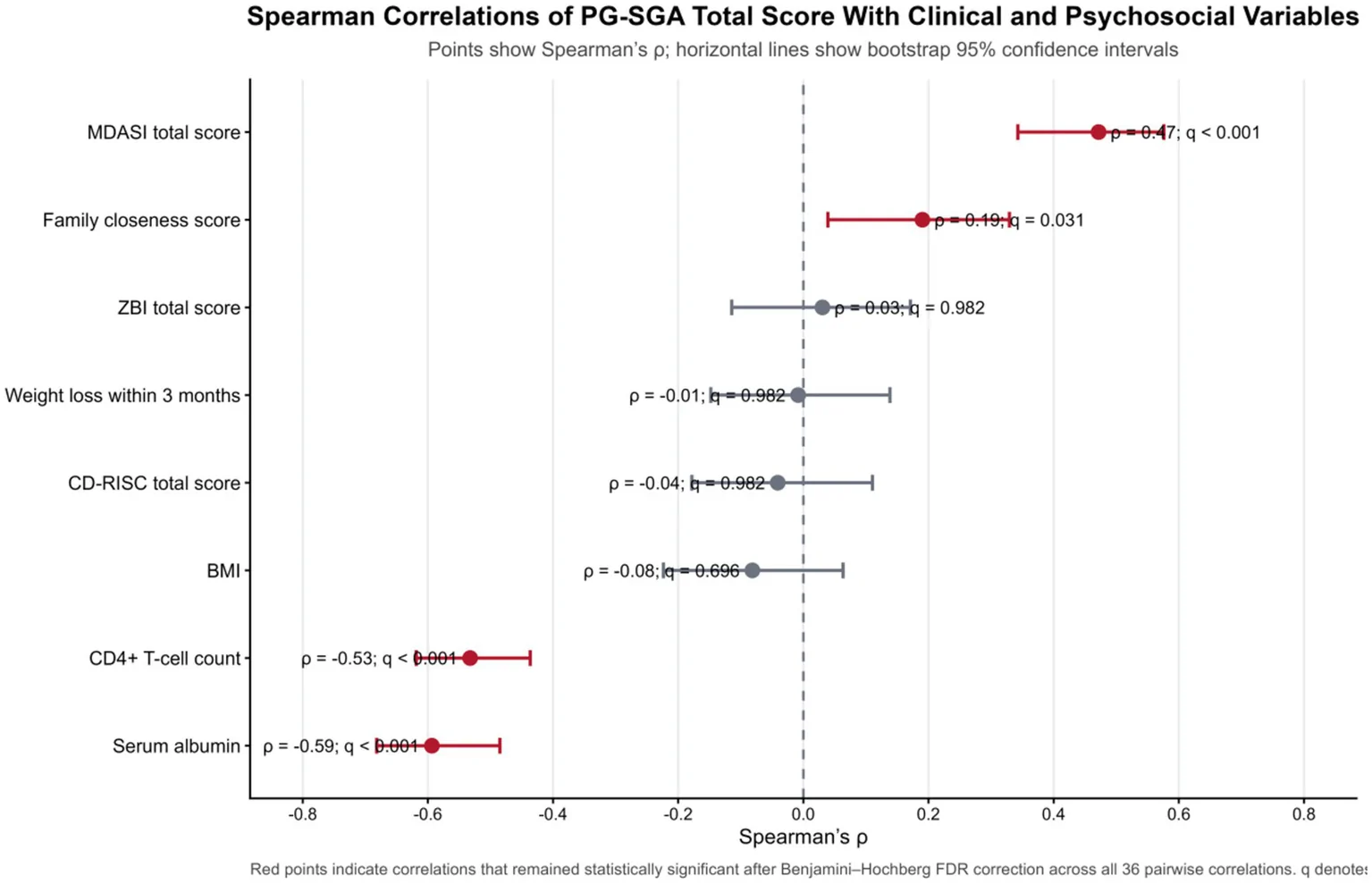
Spearman correlations of PG-SGA total score with selected clinical and psychosocial variables. Points represent Spearman rank correlation coefficients (ρ), and horizontal lines represent percentile-based 95% confidence intervals obtained from 2,000 bootstrap resamples. Red points denote correlations that remained statistically significant after Benjamini–Hochberg false discovery rate correction across all 36 pairwise comparisons; gray points denote correlations that were not statistically significant after correction. The vertical dashed line represents ρ = 0. Values adjacent to the points report the correlation coefficient and FDR-adjusted *p*-value (*q*-value). BMI, body mass index; CD4, cluster of differentiation 4; CD-RISC, Connor–Davidson Resilience Scale; FDR, false discovery rate; MDASI, MD Anderson Symptom Inventory; PG-SGA, Patient-Generated Subjective Global Assessment; ZBI, Zarit Burden Interview.

Additional statistically significant correlations included negative associations between MDASI total score and serum albumin concentration (ρ = −0.30, adjusted *p* < 0.001), MDASI total score and CD4 + T-cell count (ρ = −0.26, adjusted *p* < 0.01), and weight loss and CD-RISC total score (ρ = −0.19, adjusted *p* = 0.030). Serum albumin concentration was positively correlated with CD4 + T-cell count (ρ = 0.38, adjusted *p* < 0.001), and MDASI total score was weakly positively correlated with family closeness score (ρ = 0.18, adjusted *p* < 0.05). Selected bivariate distributions and LOESS curves are presented in Supplementary Figure S1.

Overall, the largest correlations with PG-SGA total score involved serum albumin concentration, CD4 + T-cell count, and symptom burden. These findings represent cross-sectional statistical associations and do not establish temporal direction or causal effects. The correlation between PG-SGA and MDASI should also be interpreted cautiously because the instruments contain partially overlapping symptom-related content.

### 3.4 Univariable and multivariable analyses of PG-SGA total score

Univariable associations with PG-SGA total score are presented in Table 3 and Figure 5. After Benjamini–Hochberg FDR correction across 11 variable-level tests, higher MDASI total scores, higher IPI risk, and higher family closeness scores were associated with higher PG-SGA total scores. Conversely, higher CD4 + T-cell counts and serum albumin concentrations were associated with lower PG-SGA total scores (all adjusted *p* < 0.05). Age, sex, ZBI total score, BMI, CD-RISC total score, and duration of HIV infection were not statistically associated with PG-SGA total score after FDR correction.

**Table 3:** Univariable linear regression analyses of variables associated with PG-SGA total score.

Variable	*B*	HC3 robust SE	HC3 robust 95% CI	*P-*value	FDR-adjusted *P*-value
Age, per year	0.101	0.06	−0.018 to 0.219	0.095	0.173
Female vs. male	0.639	0.926	−1.187 to 2.465	0.491	0.674
MDASI total score, per point	0.364	0.037	0.291 to 0.437	<0.001	<0.001
CD4 + T-cell count, per 100 cells/μL	−2.229	0.231	−2.685 to −1.773	<0.001	<0.001
Serum albumin, per g/L	−0.389	0.036	−0.459 to −0.318	<0.001	<0.001
IPI risk group				<0.001	<0.001
Intermediate vs. low risk	4.276	0.657	2.981 to 5.571	<0.001	
High vs. low risk	10.815	0.887	9.066 to 12.565	<0.001	
ZBI total score, per point	0.015	0.025	−0.034 to 0.064	0.544	0.674
BMI, per kg/m^2^	−0.074	0.123	−0.317 to 0.169	0.551	0.674
CD-RISC total score, per point	−0.003	0.037	−0.076 to 0.070	0.929	0.929
family closeness score, per point	0.064	0.023	0.019 to 0.109	0.006	0.012
Duration of HIV infection, per year	−0.014	0.095	−0.201 to 0.173	0.882	0.929

BMI, body mass index; CD4, cluster of differentiation 4; CD-RISC, Connor–Davidson Resilience Scale; CI, confidence interval; FDR, false discovery rate; HC3, heteroscedasticity-consistent covariance matrix estimator type 3; HIV, human immunodeficiency virus; IPI, International Prognostic Index; MDASI, MD Anderson Symptom Inventory; SE, standard error; ZBI, Zarit Burden Interview.

**Figure 5:**
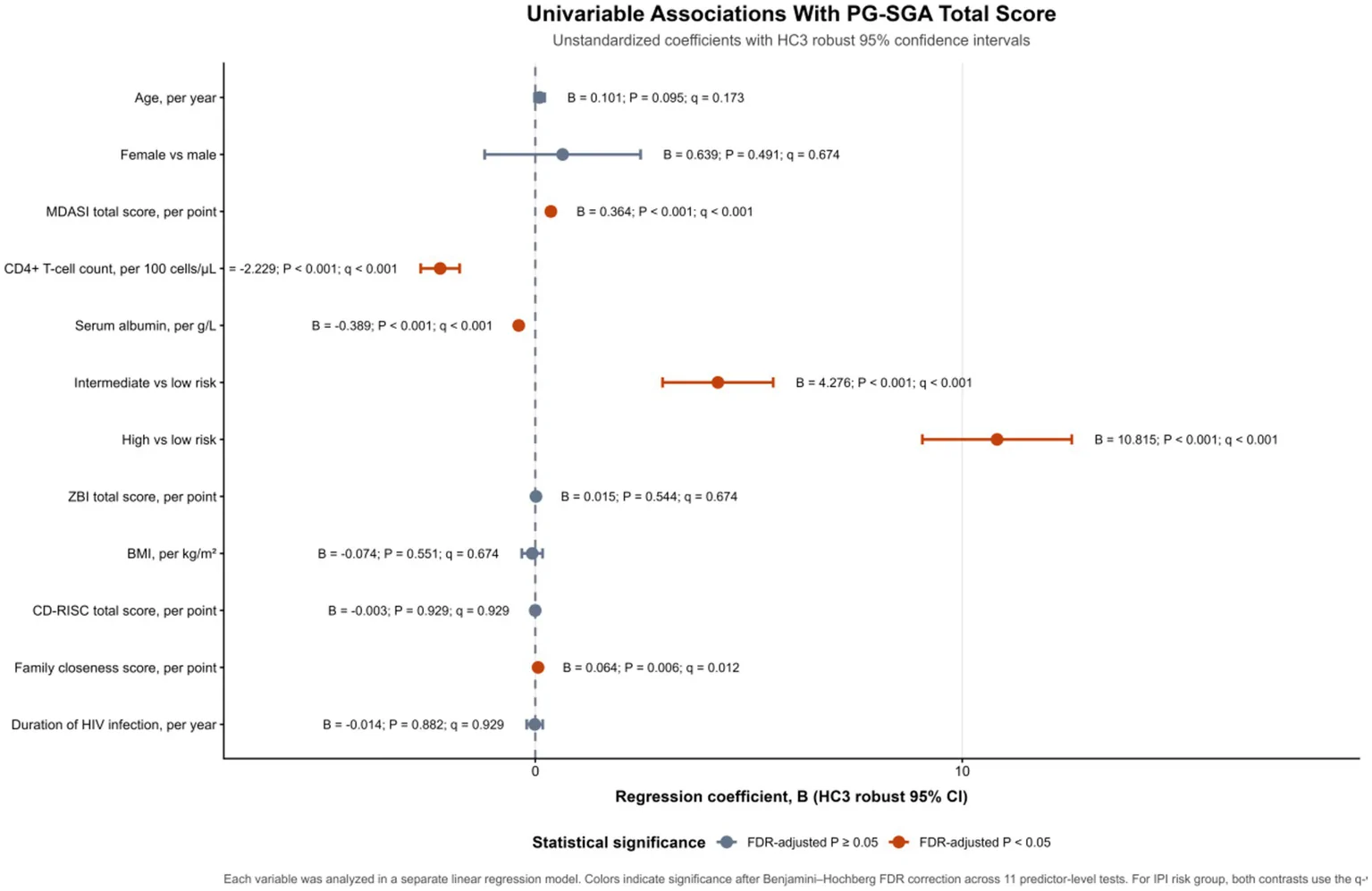
Univariable associations of sociodemographic, clinical, and psychosocial variables with PG-SGA total score. Points represent unstandardized regression coefficients (B) from separate univariable linear regression models, and horizontal lines represent HC3 heteroscedasticity-consistent 95% confidence intervals. The vertical dashed line indicates the null value (*B* = 0). Orange points and lines denote variables that remained statistically significant after Benjamini–Hochberg false discovery rate correction across 11 variable-level tests; gray points and lines denote variables that were not statistically significant after correction. Labels report B, the unadjusted *p*-value, and the FDR-adjusted *p*-value (*q*-value). For IPI risk group, both contrasts use the q-value from the overall HC3 robust Wald test. Reference categories were male sex and low IPI risk. PG-SGA grade was excluded because it was derived from the PG-SGA assessment. BMI, body mass index; CD4, cluster of differentiation 4; CD-RISC, Connor–Davidson Resilience Scale; FDR, false discovery rate; IPI, International Prognostic Index; MDASI, MD Anderson Symptom Inventory; PG-SGA, Patient-Generated Subjective Global Assessment; ZBI, Zarit Burden Interview.

In the multivariable model, MDASI total score, CD4 + T-cell count, serum albumin concentration, and IPI risk group remained statistically associated with PG-SGA total score after adjustment for age, sex, and ZBI total score (Table 4 and Figure 6). Higher MDASI scores were associated with higher PG-SGA scores (*B* = 0.233 per point, HC3 robust 95% CI: 0.159 to 0.308; *p* < 0.001). Higher CD4 + T-cell counts (*B* = −0.874 per 100 cells/μL, 95% CI: −1.304 to −0.445; *p* < 0.001) and serum albumin concentrations (*B* = −0.185 per g/L, 95% CI: −0.255 to −0.115; *p* < 0.001) were associated with lower PG-SGA scores.

**Table 4:** Multivariable linear regression analysis of variables associated with PG-SGA total score.

Variable	B	HC3 robust SE	HC3 robust 95% CI	*P*-value
Age, per year	0.054	0.041	−0.027 to 0.136	0.186
Female vs. male	0.570	0.632	−0.675 to 1.816	0.368
MDASI total score, per point	0.233	0.038	0.159 to 0.308	**<0.001**
CD4 + T-cell count, per 100 cells/μL	−0.874	0.218	−1.304 to −0.445	**<0.001**
Serum albumin, per g/L	−0.185	0.035	−0.255 to −0.115	**<0.001**
IPI risk group				**<0.001**
Intermediate vs low risk	2.784	0.991	0.829 to 4.739	**0.005**
High vs. low risk	5.208	1.259	2.725 to 7.691	**<0.001**
ZBI total score, per point	0.012	0.016	−0.019 to 0.044	0.448

*B*-values are unstandardized regression coefficients. HC3 heteroscedasticity-consistent standard errors, 95% confidence intervals, and p-values are reported because heteroscedasticity was detected. For IPI risk group, the *p*-value in the parent row refers to the overall HC3 robust Wald test. Reference categories were male sex and low IPI risk. *N* = 200; *R*^2^ = 0.588; adjusted *R*^2^ = 0.571; overall HC3 robust Wald test *p* < 0.001. PG-SGA grade was excluded because it was derived from the PG-SGA assessment. Covariates were prespecified based on clinical relevance and the conceptual framework. Given the cross-sectional design, coefficients represent adjusted statistical associations rather than causal or prospective predictive effects. CD4, cluster of differentiation 4; CI, confidence interval; HC3, heteroscedasticity-consistent covariance matrix estimator type 3; IPI, International Prognostic Index; MDASI, MD Anderson Symptom Inventory; N, number of participants included in the analysis; PG-SGA, Patient-Generated Subjective Global Assessment; R^2^, coefficient of determination; SE, standard error; ZBI, Zarit Burden Interview. Bold values indicate statistically significant associations (*P* < 0.05).

**Figure 6:**
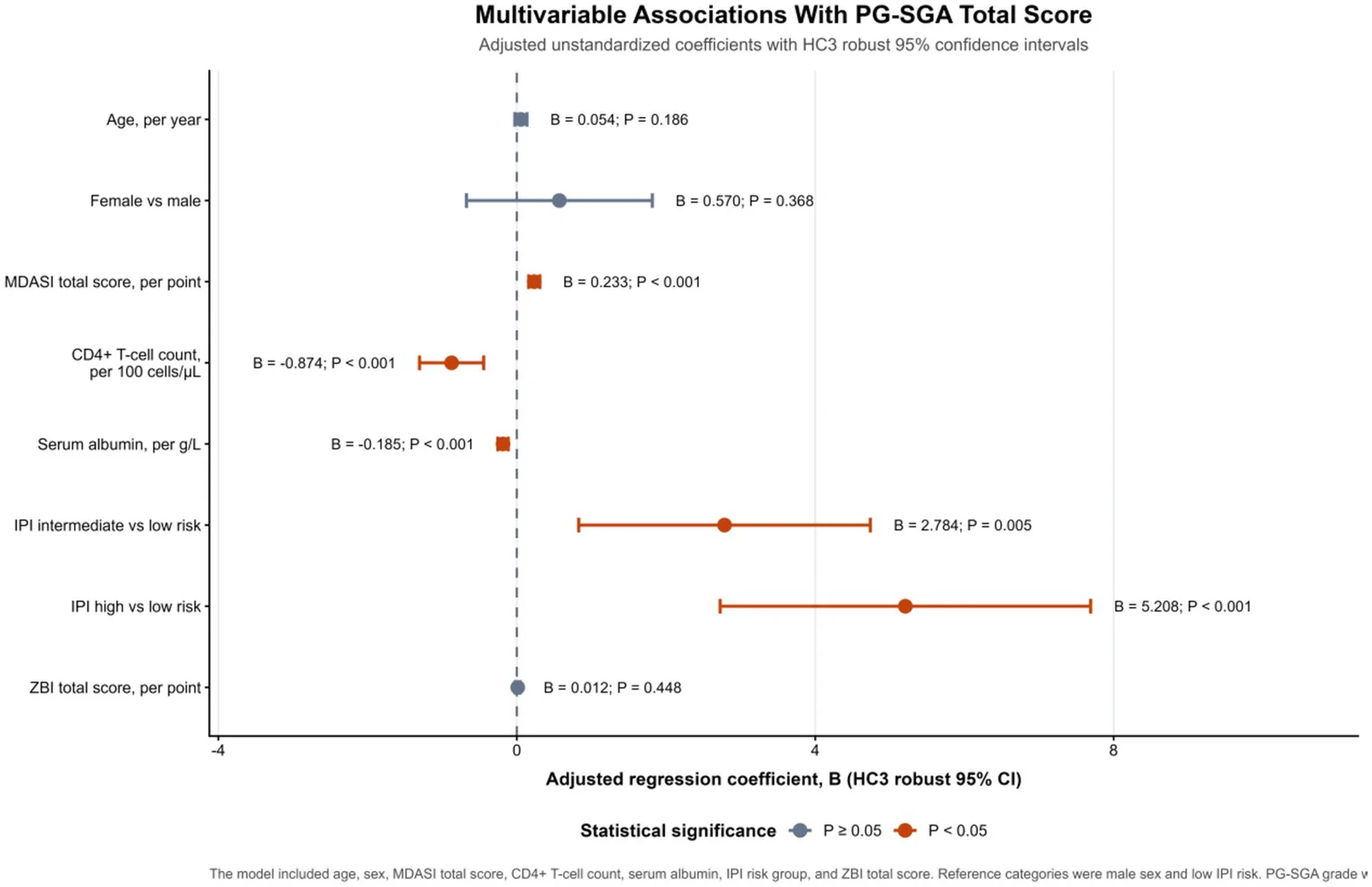
Multivariable associations of sociodemographic and clinical variables with PG-SGA total score. Points represent adjusted unstandardized regression coefficients (B), and horizontal lines represent HC3 heteroscedasticity-consistent 95% confidence intervals. The vertical dashed line indicates the null value (*B* = 0). Orange points and lines denote coefficients with HC3 robust *p* < 0.05, whereas gray points and lines denote coefficients with *p* ≥ 0.05. Labels report the adjusted coefficient and corresponding HC3 robust *p*-value. The model included age, sex, MDASI total score, CD4 + T-cell count, serum albumin concentration, IPI risk group, and ZBI total score. Reference categories were male sex and low IPI risk. PG-SGA grade was excluded because it was derived from the PG-SGA assessment. Given the cross-sectional design, the coefficients represent adjusted statistical associations and do not indicate causal or prospective predictive effects. CD4, cluster of differentiation 4; IPI, International Prognostic Index; MDASI, MD Anderson Symptom Inventory; PG-SGA, Patient-Generated Subjective Global Assessment; ZBI, Zarit Burden Interview.

IPI risk group was associated with PG-SGA total score in the overall HC3 robust Wald test (*p* < 0.001). Compared with the low-risk group, the intermediate-risk and high-risk groups had PG-SGA scores that were higher by 2.784 points (95% CI: 0.829–4.739; *p* = 0.005) and 5.208 points (95% CI: 2.725–7.691; *p* < 0.001), respectively. Age, sex, and ZBI total score were not statistically associated with PG-SGA total score in the adjusted model (all *p* > 0.05).

The multivariable model explained 58.8% of the variance in PG-SGA total score (adjusted R^2^ = 0.571), and the overall HC3 robust Wald test was statistically significant (*p* < 0.001). Regression diagnostics indicated heteroscedasticity (Breusch–Pagan *p* = 0.024) and departures from residual normality (Shapiro–Wilk *p* < 0.001; Supplementary Figure S2). These results represent unadjusted and adjusted cross-sectional associations and do not establish temporal or causal relationships.

### 3.5 Exploratory analysis of indirect statistical associations

In the covariate-adjusted parallel model, the total indirect statistical association between caregiver burden and PG-SGA total score was small and not statistically significant (*B* = 0.0117, percentile-bootstrap 95% CI: −0.0052 to 0.0294; Figure 7). None of the specific indirect associations through symptom burden, family closeness, or resilience was statistically supported because all bootstrap confidence intervals included zero (Supplementary Figure S3).

**Figure 7:**
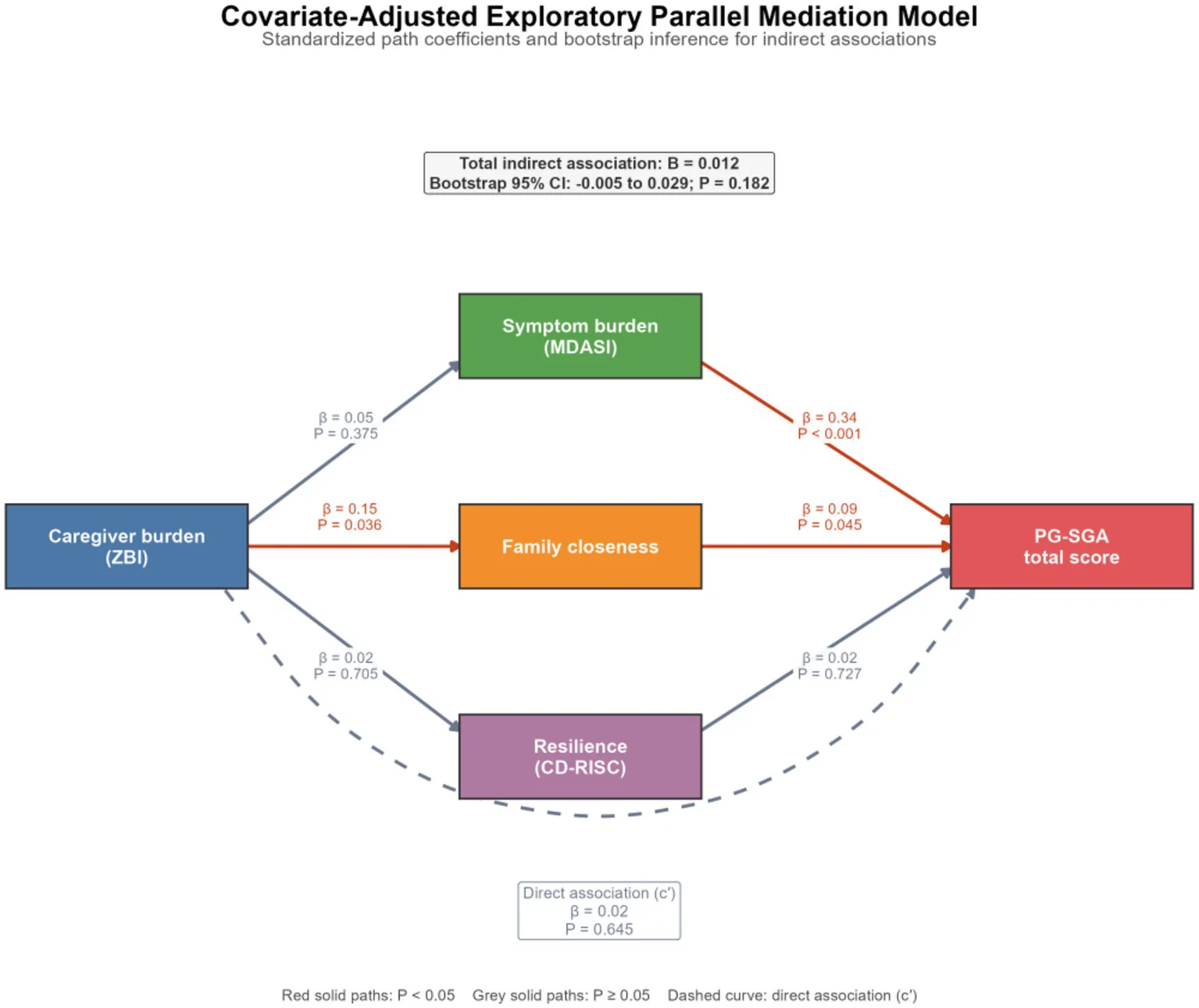
Covariate-adjusted exploratory parallel indirect-association model linking caregiver burden with PG-SGA total score. Values beside the solid arrows are standardized path coefficients (β) with corresponding *p*-values. Red solid arrows denote individual paths with *p* < 0.05, whereas grey solid arrows denote paths with *p* ≥ 0.05. The dashed curved arrow represents the direct association (c′) between caregiver burden and PG-SGA total score. The total indirect association is reported as an unstandardized coefficient (B) with a percentile-bootstrap 95% confidence interval based on 5,000 resamples. The model was adjusted for age, sex, CD4 + T-cell count, serum albumin concentration, and IPI risk group. Although some individual component paths were statistically significant, the bootstrap confidence interval for the total indirect association included zero. The model represents cross-sectional statistical associations and does not establish temporal ordering or causal mediation. ZBI, Zarit Burden Interview; MDASI, MD Anderson Symptom Inventory; CD-RISC, Connor–Davidson Resilience Scale; PG-SGA, Patient-Generated Subjective Global Assessment; IPI, International Prognostic Index.

The estimates were similar in the unadjusted and adjusted models (Supplementary Figure S4). Thus, no statistically supported indirect association was detected through the examined variables. Given the cross-sectional design, these results do not establish temporal or causal mediation and do not exclude the possibility of small associations.

### 3.6 Exploratory subgroup and sensitivity analyses

The indirect statistical association between caregiver burden and PG-SGA total score through symptom burden was examined across age, sex, and CD4 + T-cell count subgroups (Figure 8 and Table 5). All subgroup-specific bootstrap 95% confidence intervals included zero. Formal between-group comparisons also showed no differences after Benjamini–Hochberg FDR correction. The CD4 + T-cell count <200 cells/μL subgroup included only 17 participants and therefore yielded an imprecise estimate.

**Figure 8:**
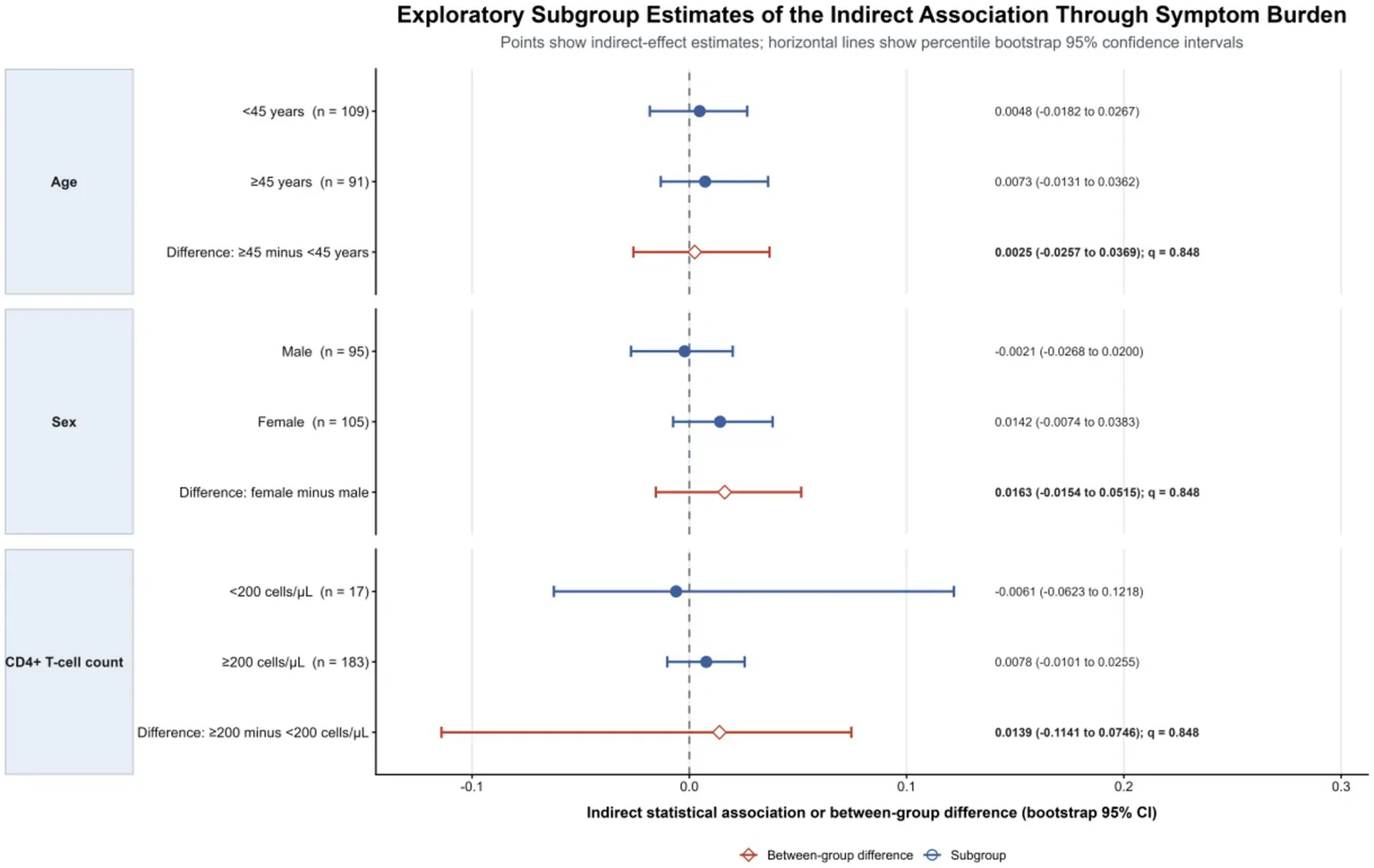
Exploratory subgroup analyses of the indirect statistical association between caregiver burden and PG-SGA total score through symptom burden. Circles represent subgroup-specific indirect-effect estimates, whereas diamonds represent formal between-group differences in indirect effects. Horizontal lines indicate percentile bootstrap 95% confidence intervals based on 5,000 resamples. The *q*-values for the three between-group comparisons were calculated using the Benjamini–Hochberg procedure. All confidence intervals included zero. The CD4 + T-cell count <200 cells/μL subgroup included only 17 participants and should be interpreted cautiously. These estimates are exploratory cross-sectional associations and do not establish mediation or causality.

**Table 5:** Exploratory subgroup analyses of the indirect statistical association between caregiver burden and PG-SGA total score through symptom burden.

Moderator	Subgroup or contrast	Estimate type	N	Indirect effect or difference	Bootstrap 95% CI	*p* value	FDR-adjusted *P* value
Age	<45 years	Subgroup-specific indirect effect	109	0.0048	−0.0182 to 0.0267		
	≥45 years	Subgroup-specific indirect effect	91	0.0073	−0.0131 to 0.0362		
	**≥45 minus <45 years**	**Between-group difference**	**109 vs. 91**	**0.0025**	**−0.0257 to 0.0369**	**0.848**	**0.848**
Sex	Male	Subgroup-specific indirect effect	95	−0.0021	−0.0268 to 0.0200		
	Female	Subgroup-specific indirect effect	105	0.0142	−0.0074 to 0.0383		
	**Female minus male**	**Between-group difference**	**95 vs. 105**	**0.0163**	**−0.0154 to 0.0515**	**0.304**	**0.848**
CD4 + T-cell count	<200 cells/μL	Subgroup-specific indirect effect	17	−0.0061	−0.0623 to 0.1218		
	≥200 cells/μL	Subgroup-specific indirect effect	183	0.0078	−0.0101 to 0.0255		
	**≥200 minus <200 cells/μL**	**Between-group difference**	**17 vs. 183**	**0.0139**	**−0.1141 to 0.0746**	**0.716**	**0.848**

Values are indirect-effect estimates or formal between-group differences in indirect effects, with percentile bootstrap 95% confidence intervals based on 5,000 resamples. For subgroup rows, *p*-values are not presented because interpretation was based on the bootstrap confidence intervals. Between-group p-values were derived from bootstrap comparisons, and FDR-adjusted p-values were calculated using the Benjamini–Hochberg procedure across the three moderator tests. All subgroup-specific and between-group confidence intervals included zero. The CD4 + T-cell count <200 cells/μL subgroup included only 17 participants and should be interpreted cautiously. These exploratory cross-sectional estimates do not establish mediation or causality. CD4, cluster of differentiation 4; CI, confidence interval; FDR, false discovery rate; N, number of participants. Bold values highlight the formal between-group difference (contrast) estimates and their associated statistics for the specified subgroups.

Sensitivity analyses using unadjusted and alternative adjusted models and excluding influential observations produced consistently small indirect-effect estimates, with all bootstrap 95% confidence intervals including zero (Supplementary Figure S5; Supplementary Table S1). Collectively, these exploratory analyses did not provide confirmatory evidence of an overall or subgroup-specific indirect statistical association through symptom burden.

## 4 Discussion

This study examined clinical, immunological, symptomatic, and caregiver related factors associated with nutritional status in patients with HIV-associated lymphoma. Higher symptom burden and IPI risk were independently associated with higher PG-SGA total scores, whereas higher CD4 + T cell counts and serum albumin concentrations were associated with lower scores. Age, sex, and caregiver burden were not independently associated with PG-SGA score. Exploratory analyses did not support an indirect association between caregiver burden and nutritional status through symptom burden or differences according to age, sex, or immune status. These findings indicate that nutritional status in this population was more consistently associated with disease related, immunological, and symptomatic characteristics than with caregiver burden.

The associations of CD4 + T cell count, serum albumin, and IPI risk with PG-SGA score are clinically plausible and consistent with previous evidence. Liu et al. found that CONUT defined nutritional status was associated with prognosis in patients with HIV-associated lymphoma, together with IPI and treatment related factors (16). A recent meta-analysis also linked PG-SGA defined malnutrition risk with adverse outcomes in oncology populations (17). Lower CD4 + T cell counts may reflect greater immune dysfunction, while serum albumin may reflect nutritional intake, inflammation, disease burden, hepatic function, and hydration. Current ESPEN and ESMO guidance therefore recommends comprehensive nutritional assessment rather than reliance on any single anthropometric or biochemical indicator (18, 19).

Symptom burden remained associated with PG-SGA score after adjustment for demographic, immunological, biochemical, prognostic, and caregiver related variables. This is consistent with recent oncology evidence linking greater symptom severity with increased malnutrition risk (20). Symptoms such as fatigue, nausea, pain, and appetite loss may coincide with reduced intake and impaired functioning. However, directionality cannot be determined: symptom burden may contribute to poorer nutrition, while malnutrition may aggravate symptoms. In addition, the PG-SGA includes nutrition-impact symptoms that overlap with domains measured by the MDASI, potentially strengthening their observed association. BMI was not associated with PG-SGA score, reinforcing the distinction between body size and multidimensional nutritional status.

Caregiver burden was not associated with PG-SGA score in the univariable or adjusted analyses, and its indirect association through symptom burden was not statistically supported. This does not demonstrate that caregiver burden is unimportant. Research in psycho-oncology indicates that caregiver burden is shaped by patient needs, family communication, social support, caregiving preparedness, and sociocultural context (21, 22). These complex relationships may not be captured by a simple cross-sectional pathway to nutritional status. Psychological resilience was also not supported as an indirect pathway, but absence of statistical significance does not prove absence of an association. Resilience may be more relevant to psychological well-being and quality of life than to nutritional status.

An important strength of this study is its focus on the understudied intersection of HIV infection, lymphoma, nutrition, and caregiving. The analysis integrated multidimensional clinical, immunological, nutritional, symptomatic, and psychosocial data from 200 patient and caregiver pairs. Nutritional status was assessed using the PG-SGA rather than inferred from BMI alone, while symptom burden, caregiver burden, and resilience were evaluated using standardized instruments. The analysis also avoided circular modelling by excluding PG-SGA grade from models of PG-SGA total score. Statistical rigor was enhanced through clinically specified covariate adjustment, HC3 robust inference, FDR correction, 5,000 resample bootstrap confidence intervals, formal between-group comparisons, and sensitivity analyses. The consistency of the principal regression findings across these analyses strengthens confidence in the observed clinical associations. Equally, the transparent reporting of small and statistically unsupported indirect associations prevents overstatement of the psychosocial findings and helps define priorities for future research.

The findings have cautious clinical implications. Symptom burden, CD4 + T cell count, serum albumin, and IPI risk may provide complementary information during nutritional evaluation. Comprehensive assessment may identify nutritional problems that would be missed by BMI alone. Caregiver and psychosocial assessments remain relevant to supportive care, but the present findings do not demonstrate that caregiver focused interventions improve nutritional outcomes. Any such benefit requires prospective evaluation.

Several limitations should be acknowledged. First, the cross sectional design prevented assessment of temporal order or causality, particularly for the assumed sequence among caregiver burden, psychosocial variables, and nutritional status, and reverse associations remain possible. Second, the single center sample limits generalizability, while differences in lymphoma subtype, disease stage, and treatment status introduced clinical heterogeneity. Third, the moderate sample size limited precision in the parallel indirect association and subgroup analyses. The CD4 + T cell count below 200 cells/μL subgroup included only 17 participants; therefore, nonsignificant findings cannot establish absence of an association. Fourth, the analyses did not fully account for ART duration and regimen, chemotherapy regimen, lymphoma treatment status, viral load, inflammatory markers, opportunistic infections, treatment toxicity, food insecurity, depression, or HIV related stigma. Fifth, self-reported psychosocial measures may be affected by reporting bias, and overlap between nutrition impact symptoms in the PG-SGA and symptoms assessed by the MDASI may have strengthened their association. Sixth, despite FDR correction, multiple exploratory comparisons increased the risks of false positive findings and insufficient power. Finally, the bounded and nonnormal PG-SGA distribution, heteroscedasticity, influential observations, and possible nonlinear patterns indicate model specification uncertainty. HC3 robust inference and sensitivity analyses improved robustness but did not eliminate these limitations.

Higher symptom burden and IPI risk were associated with poorer nutritional status, whereas higher CD4 + T cell counts and serum albumin concentrations were associated with lower PG-SGA scores. Caregiver burden was not independently associated with nutritional status, and the proposed indirect association was not confirmed. By integrating patient and caregiver data and applying robust statistical methods, this study provides a clinically grounded assessment of nutritional correlates in an understudied population. Larger multicenter longitudinal studies are needed to clarify temporal relationships and evaluate whether integrated nutritional, symptom, and psychosocial care improves patient outcomes.

## Data Availability

The raw data supporting the conclusions of this article will be made available by the authors, without undue reservation.
