# Supplementary material for: Caregiver burden and malnutrition in patients with HIV-associated lymphoma: indirect pathways via symptom distress and family closeness, and a test of resilience as a mediator
Source: Front Nutr. 2026 Sep 28;13:1921747. doi: 10.3389/fnut.2026.1921747 (PMC13654734; doi:10.3389/fnut.2026.1921747)
Supplement: Supplementary file 1 [file Supplementary_file_1.docx]

**Supplemental Figure S1. Selected bivariate associations among nutritional, clinical, and psychosocial variables.** Scatterplots show the associations between (A) PG-SGA total score and MDASI total score, (B) PG-SGA total score and CD4+ T-cell count, (C) serum albumin concentration and MDASI total score, and (D) percentage of body weight loss within the preceding 3 months and CD-RISC total score. Each point represents an individual participant. Solid curves represent locally estimated scatterplot smoothing (LOESS) fits, and shaded regions represent 95% confidence bands for the fitted curves. Each panel reports Spearman’s rank correlation coefficient (ρ) and the corresponding Benjamini–Hochberg FDR-adjusted P-value, based on correction across all 36 pairwise correlations. The LOESS curves are provided for descriptive visualization of the form of the associations and do not establish temporal or causal relationships. Abbreviations: CD4, cluster of differentiation 4; CD-RISC, Connor–Davidson Resilience Scale; FDR, false discovery rate; MDASI, MD Anderson Symptom Inventory; PG-SGA, Patient-Generated Subjective Global Assessment.

**Supplemental Figure S2. Diagnostic assessment of the multivariable linear regression model for PG-SGA total score. (A) Standardized residuals plotted against fitted PG-SGA total scores; the red line represents a locally estimated scatterplot smoothing (LOESS) curve, and the horizontal dashed line denotes zero residual. (B) Normal Q–Q plot of standardized residuals; the dashed line represents the expected distribution under normality. (C) Scale–location plot of the square root of the absolute standardized residuals against fitted values; the red line represents a LOESS curve. (D) Cook’s distances for individual observations; the horizontal dashed line denotes the screening threshold of 4/n = 0.020. Red points indicate observations exceeding this threshold, and selected observations with the largest residuals or Cook’s distances are labelled by patient ID. The plots indicated heteroscedasticity, departures from residual normality, and several potentially influential observations. These observations were retained in the primary analysis and evaluated separately in a sensitivity analysis. PG-SGA, Patient-Generated Subjective Global Assessment.**

**Supplemental Figure S3. Covariate-adjusted indirect statistical associations between caregiver burden and PG-SGA total score. Points represent unstandardized indirect-association estimates (B), and horizontal error bars represent percentile-bootstrap 95% confidence intervals based on 5,000 resamples. The vertical dashed line indicates the null value of zero. Specific indirect associations through symptom burden, family closeness, and psychological resilience, as well as their total, are presented. The model was adjusted for age, sex, CD4+ T-cell count, serum albumin concentration, and IPI risk group. All bootstrap confidence intervals included zero; therefore, none of the specific or total indirect associations was statistically significant. MDASI, MD Anderson Symptom Inventory; CD-RISC, Connor–Davidson Resilience Scale; PG-SGA, Patient-Generated Subjective Global Assessment.**

**Supplemental Figure S4. Comparison of unadjusted and covariate-adjusted indirect statistical associations between caregiver burden and PG-SGA total score. Blue circles represent estimates from the unadjusted model, and red triangles represent estimates from the covariate-adjusted model. Points indicate unstandardized indirect-association estimates (B), and horizontal error bars indicate percentile-bootstrap 95% confidence intervals based on 5,000 resamples. The vertical dashed line denotes the null value of zero. The adjusted model included age, sex, CD4+ T-cell count, serum albumin concentration, and IPI risk group. Estimates were similar before and after covariate adjustment, and all confidence intervals included zero.**

**Supplemental Figure S5. Sensitivity analyses of the indirect statistical association between caregiver burden and PG-SGA total score through symptom burden. Points represent estimated indirect effects, and horizontal lines indicate percentile bootstrap 95% confidence intervals based on 5,000 resamples. The primary adjusted model included age, sex, CD4+ T-cell count, serum albumin concentration, and IPI risk group. The expanded model additionally included BMI and duration of HIV infection. Eight observations exceeding the Cook’s distance screening threshold of 4/n were excluded in the influential-observation analysis. All confidence intervals included zero, and the findings were consistent across model specifications.**

**Supplementary Table S1. Sensitivity analyses of the indirect statistical association between caregiver burden and PG-SGA total score through symptom burden**

| **Analysis** | **N** | **Indirect effect** | **Bootstrap 95% CI** | **Bootstrap P-value** | **Valid bootstrap repetitions** |
| --- | --- | --- | --- | --- | --- |
| **Primary adjusted model** | **200** | **0.0069** | **-0.0082 to 0.0236** | **0.378** | **5,000** |
| **Unadjusted model** | **200** | **0.0076** | **-0.0183 to 0.0336** | **0.558** | **5,000** |
| **Expanded covariate adjustment** | **200** | **0.0071** | **-0.0085 to 0.0231** | **0.368** | **5,000** |
| **Excluding influential observations** | **192** | **0.0028** | **-0.0123 to 0.0191** | **0.704** | **5,000** |

**Indirect effects and percentile bootstrap 95% confidence intervals were estimated using 5,000 resamples. The primary adjusted model included age, sex, CD4+ T-cell count, serum albumin concentration, and IPI risk group. The expanded model additionally included BMI and duration of HIV infection.**

**Influential observations were screened using Cook’s distance >4/n; eight observations were excluded in that sensitivity analysis. All bootstrap 95% confidence intervals included zero. Estimates represent exploratory cross-sectional indirect statistical associations and do not establish mediation or causality.**
